# Novel anti-CEA affibody for rapid tumor-targeting and molecular imaging diagnosis in mice bearing gastrointestinal cancer cell lines

**DOI:** 10.3389/fmicb.2024.1464088

**Published:** 2024-10-09

**Authors:** Huanyi Shao, Kaiji Lv, Pengfei Wang, Jinji Jin, Yiqi Cai, Jun Chen, Saidu Kamara, Shanli Zhu, Guanbao Zhu, Lifang Zhang

**Affiliations:** ^1^Department of Pediatric Surgery, The First Affiliated Hospital of Wenzhou Medical University, Wenzhou, China; ^2^Department of Microbiology and Immunology, School of Basic Medical Sciences, Institute of Molecular Virology and Immunology, Wenzhou Medical University, Wenzhou, China; ^3^Department of Gastrointestinal Surgery, The First Affiliated Hospital of Wenzhou Medical University, Wenzhou, China

**Keywords:** affibody molecules, gastrointestinal cancer, CEA, molecular imaging, tumor diagnosis

## Abstract

Gastrointestinal cancer is a common malignant tumor with a high incidence worldwide. Despite continuous improvements in diagnosis and treatment strategies, the overall prognosis of gastrointestinal tumors remains poor. Carcinoembryonic antigen (CEA) is highly expressed in various types of cancers, especially in gastrointestinal cancers, making it a potential target for therapeutic intervention. Therefore, the expression of CEA can be used as an indication of the existence of tumors, chosen as a target for molecular imaging diagnosis, and effectively utilized in the targeted therapy of gastrointestinal cancers. In this study, we report the selection and characterization of affibody molecules (Z_CEA_539, Z_CEA_546, and Z_CEA_919) specific to the CEA protein. Their ability to bind to recombinant and native CEA protein has been confirmed by surface plasmon resonance (SPR), immunofluorescence, and immunohistochemistry assays. Furthermore, Dylight755-labeled Z_CEA_ affibody showed accumulation within the tumor site 1 h post injection and was continuously enhanced for 4 h. The Dylight755-labeled Z_CEA_ affibody exhibited high tumor-targeting specificity in CEA+ xenograft-bearing mice and possesses promising characteristics for tumor-targeting imaging. Overall, our results suggest the potential use of Z_CEA_ affibodies as fluorescent molecular imaging probes for detecting CEA expression in gastrointestinal cancer.

## 1 Introduction

Gastrointestinal cancers are one of the five most common cancers globally. Among them, stomach and colorectal cancers have a high rate of morbidity (25.9 per 100,000; 22.4 per 100,000) and mortality (11.8 per 100,000; 14.6 per 100,000) in eastern Asia, especially in China, Japan, and South Korea (Sung et al., 2021). Although recent advancements and the widespread use of endoscopy have improved the early diagnosis of gastrointestinal tumors, it is still missed as it heavily depends on physicians’ clinical experience and skills (Axon, 2008; Zhang et al., 2016). Surgical resection is the main course of treatment for patients with gastrointestinal tumors. The identification and confirmation of tumor sites and lymph node metastases during surgery mainly depends on the subjective judgment of the doctor. An imprecise judgment has a great impact on the effect of surgical treatment, which may lead to recurrence and metastasis after surgery (Shah and Weissleder, 2005). The current challenges in the diagnosis and surgical treatment may be associated with the poor prognosis of gastrointestinal tumors (Allemani et al., 2018; Wang et al., 2023; Schlick et al., 2020). Therefore, the improvement of diagnostic and surgical accuracy is urgently needed to improve early diagnosis and clinical outcomes for patients. Imaging techniques such as fluorescent molecular imaging can help clinicians display tumor lesions in endoscopic operations and surgeries, enhancing surgical visibility.

Carcinoembryonic antigen (CEA) is a tumor-associated antigen that was initially discovered in 1965 from fetal colon tissue (Gold and Freedman, 1965). CEA is a glycosylated cell surface protein that belongs to the immunoglobulin superfamily adhesion molecules, with a molecular weight of approximately 180–200 kDa, containing a N-terminal Ig V-like domain (N-domain), three Ig-like C2-domains, and a glycophosphatidylinositol (GPI)-membrane-anchored membrane protein (Zimmermann et al., 1987; Thompson et al., 1987; Beauchemin et al., 1987; Hefta et al., 1988). The non-regulated overexpression of CEA plays an important role in the development of many cancer cells, including inhibiting cell loss and apoptosis (Ordoñez et al., 2000), destroying cell polarization and tissue structure (Ilantzis et al., 2002), and inhibiting differentiation procedures (Ilantzis et al., 2002; Eidelman et al., 1993). CEA can also be used as a homotypic or heterotypic adhesion molecule, or in combination with signaling receptors such as DR5 receptors and transforming growth factor-β receptors, which can affect tumor cells or the surrounding interstitial and immune compartments to change their signaling programs to support metastatic progress (Beauchemin and Arabzadeh, 2013). The CEA, which is frequently overexpressed in various gastrointestinal cancers, has become a promising target for tumor diagnostics and targeted therapies (Beauchemin and Arabzadeh, 2013; Nolan et al., 1999).

Affibody molecules are a new class of small (~6.5 KDa) affinity proteins derived from the immunoglobulin G (IgG) binding region of *Staphylococcus aureus* protein A (SPA) (Uhlén et al., 1984). Based on randomized combination of 13 amino acid residues located within Z-domain scaffold of the IgG-binding region, large libraries can be constructed, from which potent binders can be screened to bind to any given target molecule with high affinity and specificity (Nord et al., 1995; Ståhl et al., 2017). Affibodies have similar binding characteristics and capabilities to antibodies. They possess some unique advantages over antibodies, such as stable physical and chemical properties, fast tumor localization, rapid clearance from blood, and low immunogenicity, making the affibody molecules extremely attractive for many medical applications, including *in vivo* molecular imaging, receptor signal blocking, and biotechnology applications (Ståhl et al., 2017). To date, more than 500 published studies related to this topic have revealed that affibody molecules targeting approximately 50 different proteins have been selected and used as high-affinity moieties in various medical applications (Zhu et al., 2021). These affibody molecules targeted proteins such as human epidermal growth factor receptor 2 (HER-2) (Baum et al., 2010), vascular endothelial growth factor (VEGF) (Fedorova et al., 2011), epidermal growth factor receptor (EGFR) (Garousi et al., 2016), human epidermal growth factor receptor 3 (HER3) (Schardt et al., 2017), and Epstein–Barr virus latent membrane proteins (EBV LMP1 and LMP2) (Zhu J. et al., 2020; Zhu S. et al., 2020). Affibody molecules have become promising agents for molecular imaging detection and targeted tumor therapy.

In this study, we report the selection and characterization of affibody molecules specific to the CEA protein. By biopanning, enzyme-linked immunosorbent assay (ELISA)-based screening, and DNA sequencing, three potential affibody molecules were obtained (Z_CEA_539, Z_CEA_546, and Z_CEA_919) from a phage display library. Their ability to bind to recombinant and native CEA protein have been confirmed by surface plasmon resonance (SPR), immunofluorescence, and immunohistochemistry assays. Furthermore, Dylight755-labeled Z_CEA_ affibody showed accumulation within the tumor site 1 h post injection and was continuously enhanced for 24 h. The Dylight755-labeled Z_CEA_ affibody exhibited high tumor-targeting specificity in CEA+ xenograft-bearing mice and also possesses promising characteristics that make them suitable for tumor-targeting imaging. We hypothesized that the generation of CEA-binding affibody molecules could accelerate the diagnosis and treatment of gastrointestinal cancer.

## 2 Materials and methods

### 2.1 Predicting immunodominant epitopes of CEA

The complete amino-acid sequence of CEA was acquired from the UniprotKB/Swiss-Prot database. The secondary structure of CEA amino acid sequence was analyzed by Garnier–Osguthorpe–Robson (GOR4) (Garnier et al., 1996), Self-Optimized Prediction Method (SOPMA) (Geourjon and Deléage, 1995), and Protein Secondary Structure (PSS) provided on the EXPASY server. Hydrophilicity, polarity, flexibility, accessibility, and antigenicity of CEA protein were analyzed by the methods of Hopp and Woods (1981), Zimmerman et al. (1968), Emini et al. (1985), and Jameson and Wolf (1988), respectively. Transmembrane domains were analyzed by TransMembrane prediction using Hidden Markov Models (TMHMM). Combined with the prediction results, the antigenicity index established by Jameson and Wolf (1988) was used to comprehensively evaluate the immunodominant B-cell epitopes of CEA and preliminarily select the target peptide for screening. Subsequently, the target peptide was analyzed with the NetCTL 1.2 webserver to find the dominant cytotoxic T-lymphocyte (CTL) epitope.

### 2.2 Construction of phage display library of the Z domain from staphylococcal protein A

A phage display library was constructed as described previously (Xue et al., 2016). Affibody proteins were selected from a phage display combinatorial library containing 13 randomized amino acid residues in helices 1 and 2 of the Z domain. A wild SPA-Z scaffold was used as a template for polymerase chain reaction (PCR) with the random primers designed based on the corresponding sequences to the helical regions. Then, the gene fragments were digested by restriction endonucleases *Sfi I* and *Not I* (Thermo Fisher Scientific, Boston, MA) and cloned to the phagemid vector (pCANTAB5E) to construct the recombinant phagemid vector (pCANTAB5E/SPA-N). The recombinant phagemids were transformed into competent *Escherichia coli* TG1 cells, with a library complexity of 1 × 10^9^ and 100% diversity in SPA-Z scaffold. Then, for an assessment of the affibody library capacity, phage stocks were resuspended and stored in sterile phosphate-buffered saline (PBS)/glycerol (20% v/v) at −80°C.

### 2.3 Phage display selection of potential affibody molecules targeting CEA

A synthetic peptide (Shanghai Bootech BioScience & Technology Co., Ltd., Shanghai, China) with the dominant immune epitope of CEA 148–175 amino acids was used as a target for three rounds of biopanning and enzyme-linked immunosorbent assay (ELISA). In brief, phage selection of the binders to the CEA (148–175 amino acids) peptide was performed in an immunotube as previously described (Aavula et al., 2011). ELISA was used to further measure their binding affinities to the target peptide as described by Xue et al. (2016). After DNA sequencing, the sequences of the inserted fragments in the selected phages were designated as potential affibodies with high binding affinity and selectivity to CEA recombinant protein.

### 2.4 Expression and purification of CEA-binding affibody molecules

The DNA sequences encoding the selected CEA-binding affibody molecules (Z_CEA_539, Z_CEA_546, and Z_CEA_919) and wild SPA-Z scaffold (Z_WT_) were subcloned into *E. coli* expression vector *pET21a* (+) with the addition of His6 tag. The recombinant plasmids were transformed into *E. coli* BL21(DE3) and induced by 1 mM isopropyl *β*-D1- thiogalactopyranoside (IPTG, Sigma-Aldrich, St. Louis, MO) for protein expression. Recombinant proteins were purified by affinity chromatography using Ni-NTA Sepharose column (Qiagen, Hilden, Germany) and were dialyzed for 2 h in PBS using Slide-A-Lyzer (Pierce, Rockford, IL). The molecular weight and purity of the fusion proteins were confirmed by sodium dodecyl sulfate-polyacrylamide gel electrophoresis (SDS-PAGE) and Western blotting using an anti-His-tag monoclonal antibody (MultiSciences Biotech Co., Ltd., Hangzhou, China). The concentrations of the proteins were determined by the bicinchoninic acid (BCA) kit (Beyotime, Beijing, China) and protein quantitation method. The purified proteins were aliquoted and stored at −20°C.

### 2.5 Surface plasmon resonance

To analyze the biospecific interaction between the selected affibody molecules and CEA protein, surface plasma resonance (SPR) was performed on a Biacore T200 (GE Healthcare, Uppsala, Sweden). The full-length CEA protein serving as a ligand was successfully immobilized onto a sensor chip CM5 (GE Healthcare) according to the manufacturer’s instructions. Then, four different concentrations were prepared (5, 2.5, 1.25, and 0.625 μM) and injected over the immobilized sensor chip (GE Healthcare) surface to monitor the protein–ligand interaction. SPR data were determined using the 1:1 binding model in Biacore T200 evaluation software.

### 2.6 Cell culture

The CEA+ cell lines HT-29, MKN-45, and CEA-HeLa229 were bought from American Type Culture Collection (ATCC). These cell lines (HT-29 and MKN-45) express high levels of CEA and were used to study Z_CEA_ binding selectivity toward the target protein. All cell lines were cultured in either RPMI-1640 medium or Dulbecco’s modified eagle medium (DMEM), supplemented with 10% fetal bovine serum and antibiotics (penicillin-streptomycin100 units/mL and 0.1 mg/mL) that were bought from Gibco. According to the supplier’s recommendation, the number of passages for cell lines were limited to five to eight passages after purchase.

### 2.7 Immunofluorescence assay

To assess the targeting specificity of Z_CEA_ affibodies *in vitro*, an indirect immunofluorescence analysis was performed. In brief, HT-29, MKN-45, and HeLa229 cells were seeded in a 24-well plate at a density of 2 × 10^3^ cells/well, stored in 5% CO_2_, and incubated at 37°C. Then, the cells were incubated with 100 μg/mL of Z_CEA_ affibodies or Z_WT_ for 3 h. The cells were washed with PBS, fixed with 4% paraformaldehyde, permeabilized in PBS containing 0.3% Triton X-100 (Sigma-Aldrich, St. Louis, MO) for 10 min at 37°C, blocked with blocking buffer for 2 h, and then incubated with mouse anti-His monoclonal antibody at 4°C overnight. Subsequently, the cells were stained for 1 h at 37°C with FITC-conjugated goat anti-mouse IgG (H + L). Cell nuclei were counterstained with Hoechst 33342 (blue) (Beyotime Biotech Co. Ltd., China), and the images were visualized using a confocal fluorescence microscope (Nikon C1-i, Japan).

### 2.8 Immunohistochemical staining

A variety of human gastric tissues (three to five cases) were obtained from surgically removed specimens, fixed with 4% formalin for 24 h, embedded in paraffin, and stored at 4°C. All experimental protocols were reviewed and approved by the Ethical Committee of the First Affiliated Hospital of Wenzhou Medical University. After routine deparaffinization and rehydration, slides were immersed in 3% H_2_O_2_ for 10 min, and the antigen was retrieved by heating the slides in 0.01 M sodium citrate buffer (pH 6.0) at 96°C for 15 min. The slides were blocked with 5% normal goat serum (Cell Signalling Technology) for 1.5 h at 37°C. Then, the slides were probed with affibodies at 4°C overnight and were incubated with the anti-His monoclonal antibody for 0.5 h at 37°C, followed by horse radish peroxidase (HRP)-conjugated goat-anti mouse antibody for 0.5 h at 37°C. Polyclonal rabbit anti-CEA serum (prepared in-house) was used as a positive control. Finally, the slides were stained with DAB and counterstained with hematoxylin.

### 2.9 Biodistribution of CEA-binding affibody molecules in tumor xenograft mice

The *in vivo* biodistribution and targeting ability of Z_CEA_ affibody was detected by near-infrared fluorescence imaging. Female Balb/c nude mice aged 4- to 6-weeks and purchased from the Nanjing Biomedical Research Institute in China were used to establish the HT-29, MKN-45, and HeLa229 mouse xenograft models. Then, we subcutaneously injected the nude mice in the right forearm region with 1 × 10^7^ cells/100 μL PBS (*n* = 5 per group). When the tumor volume reached approximately 200–500 mm^3^, mice were taken for imaging. DyLight755 (Thermo Fisher Scientific) has an excitation peak of 754 nm and an emission peak of 776 nm, which was used to label Z_CEA_546 and Z_WT_ affibody according to the manufacturer’s instructions. Then, 100 μmol of Dylight-755-labeled Z_CEA_546 or Z_WT_ dissolved in 150 μL PBS were injected intravenously through the tail vein under brief anesthesia (1.4% isoflurane for 5 min) and imaged using NIR imaging system (CRi Maestro 2.10, Perkin Elmer, Waltham, MA) at different time points (5 min, 30 min, 1 h, 1.5 h, 2 h, 4 h, 6 h, 8 h, and 24 h). After injection, tumor/skin tissue signal intensity was analyzed at different time points using GraphPad Prism software. Then, the tumor-bearing mice were sacrificed to collect the main organs such as the heart, liver, spleen, lung, and kidney, and their fluorescence intensity were measured as describe above.

### 2.10 Statistical analysis

Two-tailed, single Student’s t-tests were performed to determine statistical significance between groups, with *p* < 0.05 denoting statistical significance. All graphs were acquired using GraphPad Prism.

## 3 Results

### 3.1 Expression and purification of Z_CEA_ affibodies

**Figure 1:**
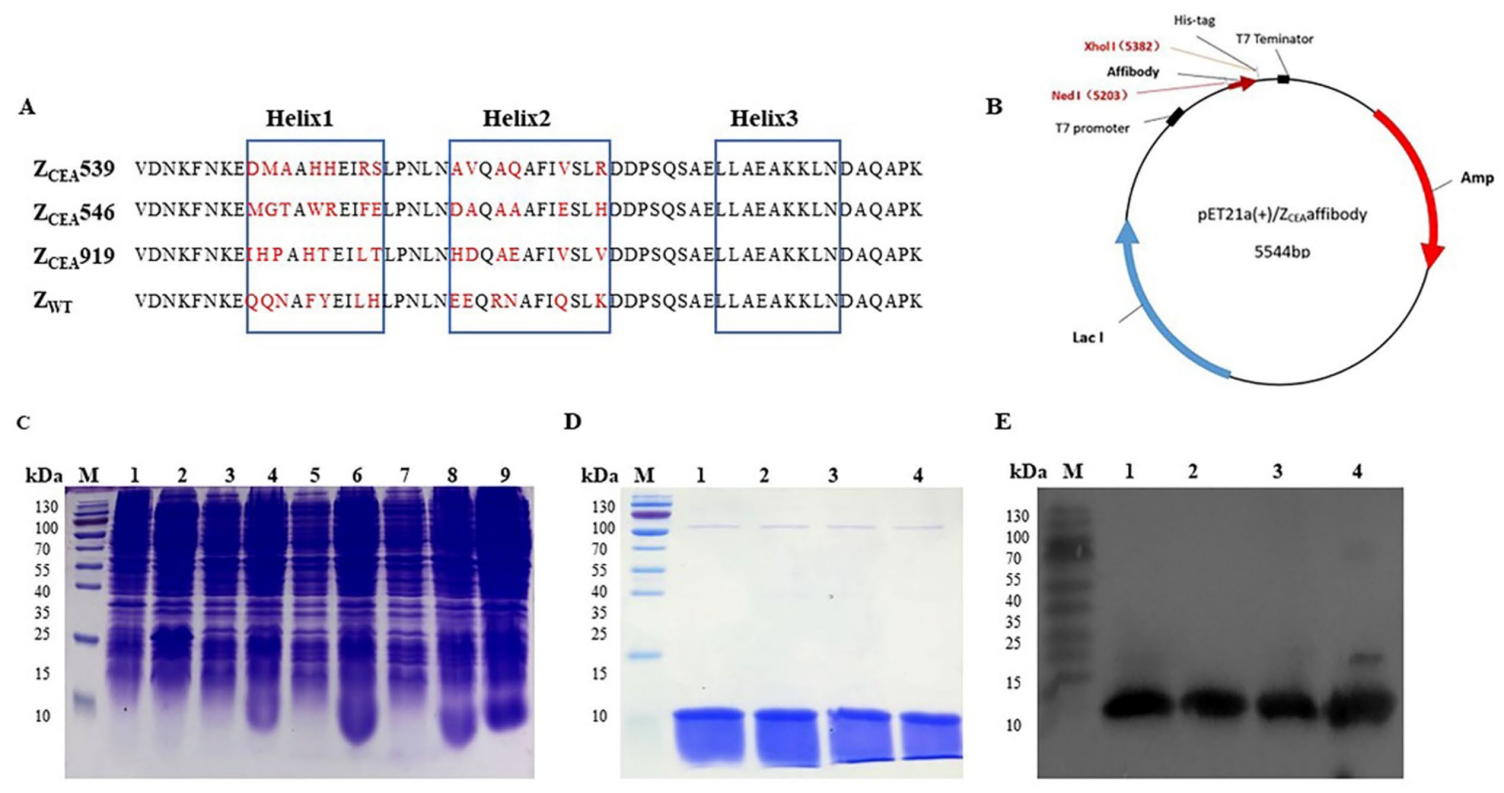
Expression, purification, and detection of Z_CEA_ affibodies. **(A)** Amino acid sequence alignment of Z_CEA_ affibodies and Z_WT._ The three blue boxes are the α-helices subdomain and the randomized amino acid is indicated in red. **(B)** Schematic structure of the pET21a (+)/Z_CEA_ recombinant plasmid. **(C)** Protein analysis by SDS-PAGE stained with Coomassie brilliant blue. Lane M, protein marker; lane 1, empty *E. coli* BL21; lane 2, empty vector of *E. coli* BL21; (3, 5, 7), total protein before IPTG induction, and lane (4, 6, 8, 9), after IPTG induction. **(D)** Lane M, protein marker, lane 1, Z_CEA_539; lane 2, Z_CEA_546; lane 3, Z_CEA_919, and lane 4, Z_WT_. **(E)** Lane M, protein marker, lane 1, Z_CEA_539; lane 2, Z_CEA_546; lane 3, Z_CEA_919, and lane 4, Z_WT_. Experiments were performed in triplicate.

Three cycles of panning were performed to select the positive clones from the phage display library. Then the phage clones were screened using ELISA to identify high-affinity Z_CEA_ binding clones and were analyzed through DNA sequencing. Through sequencing, we were able to identify a total of 31 clones (31/120 or 25.8%). Three potential Z_CEA_ affibodies (Z_CEA_539, Z_CEA_546, and Z_CEA_919) were highly expressed and purified. This affibody framework region is highly homologous but diverse in the helical regions (Figure 1A). The affibody DNA sequences were successfully inserted into the expression vector pET21a (+) with His6 tag fusion protein (Figure 1B). Then, the recombinant plasmids were transformed into *E. coli* BL21(DE3) and were induced by IPTG for protein expression (Figure 1C). The expressed proteins were detected by SDS-PAGE analysis (Figure 1D). Western blotting further confirmed that the fusion proteins could react with the monoclonal antibody specific to six histidine tags (Figure 1E).

### 3.2 Analysis of Z_CEA_ protein–CEA binding affinity

**Figure 2:**
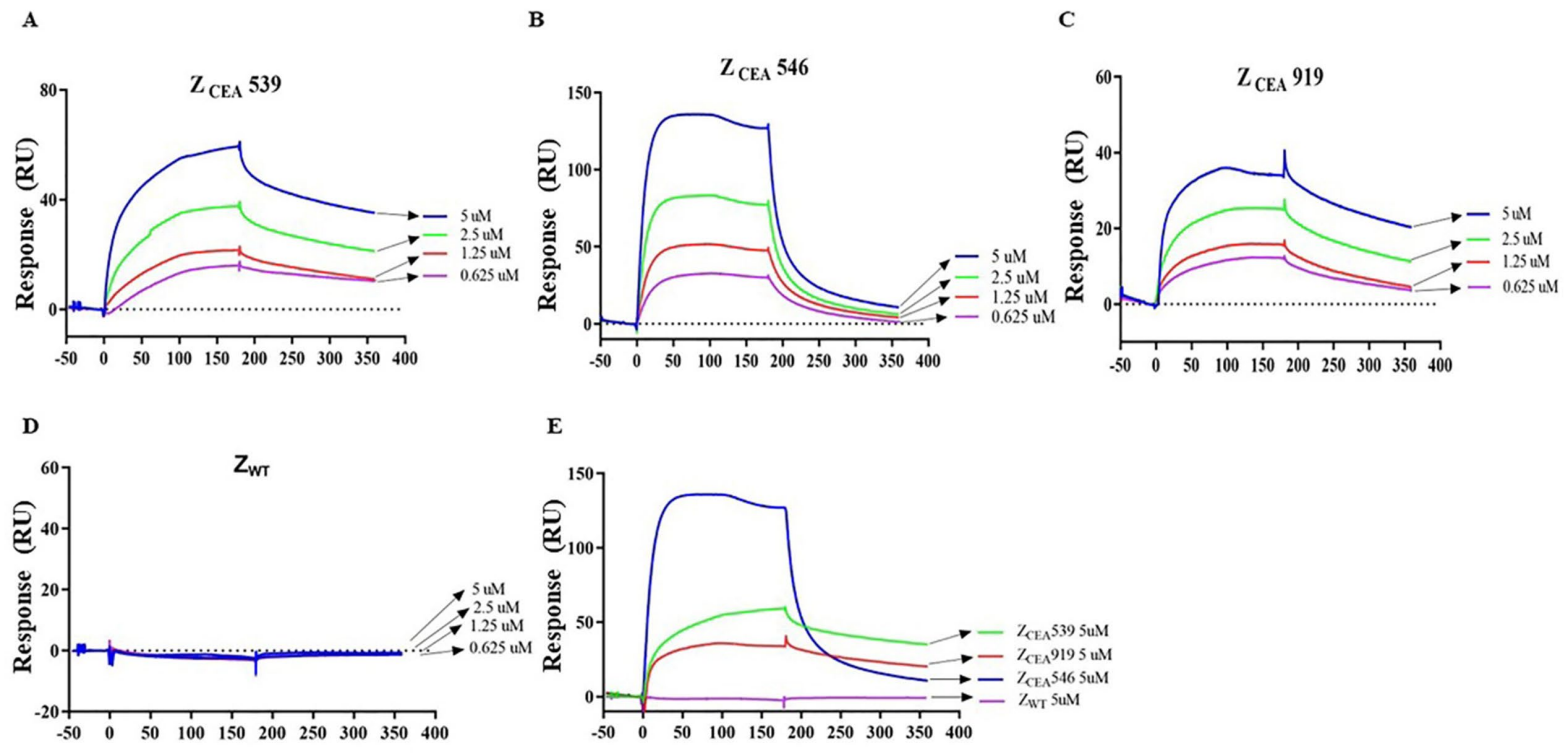
Measuring Z_CEA_ affibodies-CEA binding kinetics using SPR. **(A–C)** Sensograms obtained from different concentrations injected over the immobilized sensor chip surface to monitor Z_CEA_ affibody–CEA interaction. **(D)** Z_WT_ served as a control group. **(E)** Sensogram obtained for the highest concentration (5 μM) of Z_CEA_ affibodies and Z_WT_. Experiments were performed in triplicate.

SPR studies were carried out to measure the binding affinity of Z_CEA_539, Z_CEA_546, and Z_CEA_919 to the target protein CEA using BIAcore T200 biosensor. Z_CEA_ affibodies of various concentrations (5, 2.5, 1.25, and 0.625 μM) were prepared to flow over the immobilized CEA target protein. Our results demonstrated a concentration-dependent increase in resonance signals and strong binding affinity of three selected Z_CEA_ affibodies to ligand (Figures 2A–C). In contrast, Z_WT_ did not show the protein–ligand binding interaction (Figure 2D). The sensogram shows the maximum concentration (5 μM) of ZCEA affibodies and Z_WT_ (Figure 2E). We then used BIAcore analysis software 3.0 (Biacore) to measure the binding kinetics of Z_CEA_ protein–ligand interaction, and sensograms were fitted to a 1:1 Langmuir model. The equilibrium dissociation constant (KD) for the binding of Z_CEA_539, Z_CEA_546, Z_CEA_919, and Z_WT_ were 5.059 × 10^−6^ mol/L, 1.733 × 10^−7^ mol/L, 5.512 × 10^−6^ mol/L, and 1.628 mol/L, respectively (Supplementary Table S1). Our SPR results showed a strong high-affinity binding of Z_CEA_ affibodies to CEA target protein, while Z_WT_ did not display any binding interaction toward the target protein.

### 3.3 *In vitro* tumor cell binding

**Figure 3:**
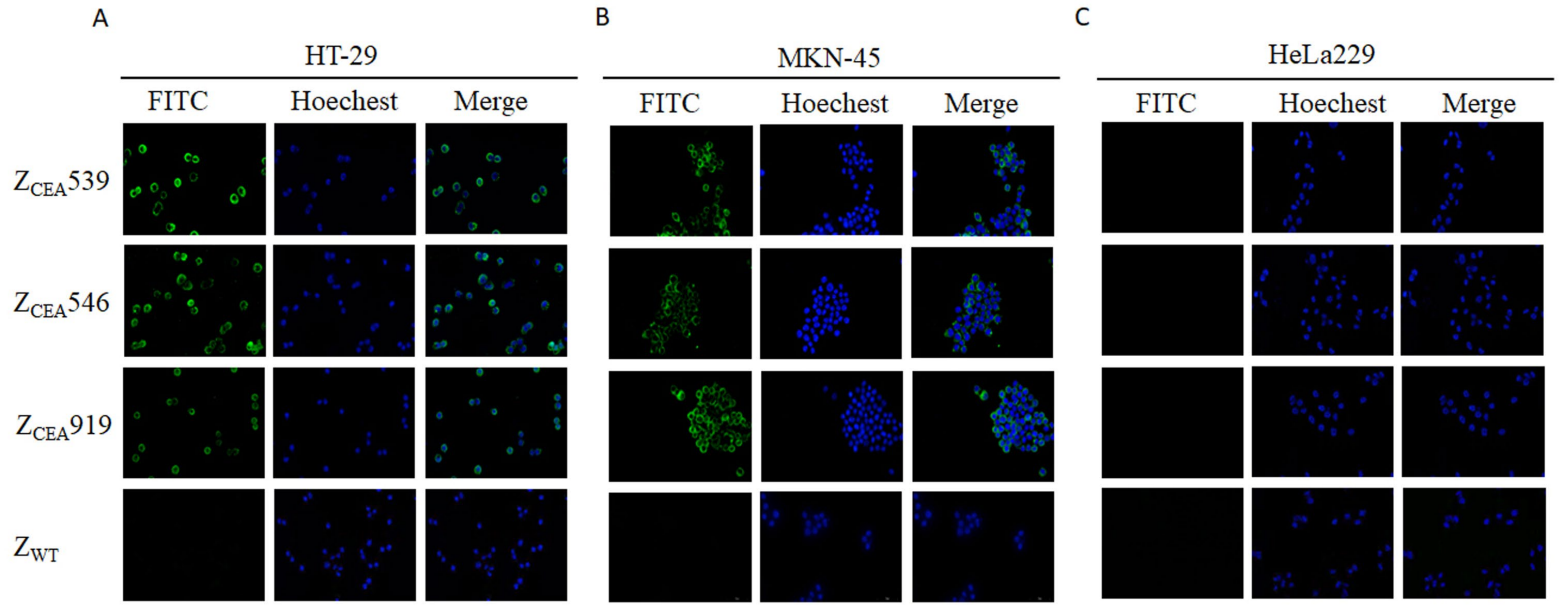
Binding of Z_CEA_ affibodies to HT-29 and MKN-45 cells by indirect immunofluorescence assay. **(A)** HT-29 **(B)** MKN-45 cells were incubated with Z_CEA_ affibodies. Mouse-anti-His-tag mAb FITC (green) was used to visualize the binding of Z_CEA_ affibodies to CEA+ cancer cell lines and nuclei were counterstained with Hoechst 3342 (400×). **(C)** HeLa229 served as the CEA cancer cell line. Z_WT_ set as the affibody negative control. Scale bar = 10 μm. Experiments were performed in triplicate.

Immunofluorescence assays were employed to further confirm the specific binding of Z_CEA_ affibodies to tumor cells that highly express CEA protein. Bright green fluorescence staining was clearly observed at the cell membrane region of CEA+ cell lines (HT-29 and MKN-45) that were treated with Z_CEA_ affibodies (Figures 3A,B), while no bright green fluorescence staining was noticed in cells treated with Z_WT_. Furthermore, CEA cell line (HeLa229) treated with Z_CEA_ affibodies or Z_WT_ did not show any fluorescence signal (Figure 3C). This result further confirms that Z_CEA_539, Z_CEA_546, and Z_CEA_919 affibodies have strong binding affinity and specificity to target protein (CEA) expressed in HT-29 and MKN-45 cell lines.

### 3.4 Immunohistochemistry

**Figure 4:**
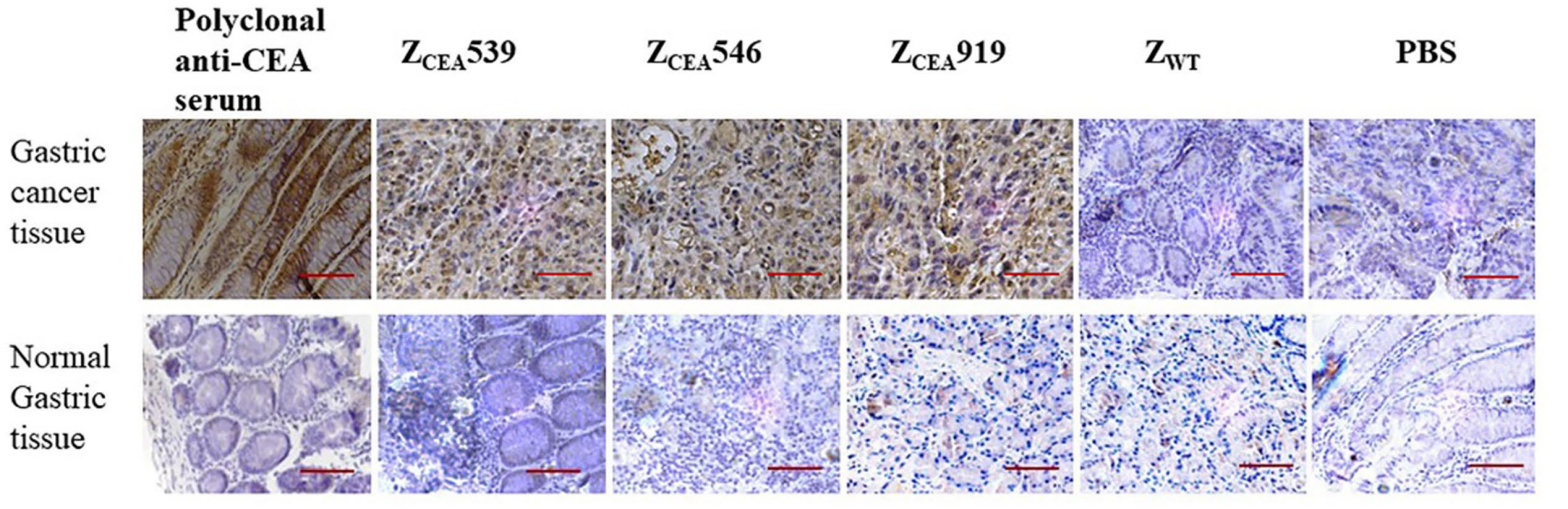
Representative images of hematoxylin and eosin (HE) and immunohistochemical expression of CEA in gastric cancer tissue and normal gastric tissue. Gastric cancer tissue (upper panel) and normal gastric tissue (lower panel) were stained with Z_CEA_ affibodies. Polyclonal anti-CEA as positive control; Z_WT_ and PBS as negative controls. Magnification 400×. Scale bar = 50 μM. Experiments were performed in triplicate.

Histological analysis of the tumor tissue was performed to further investigate the binding specificity of Z_CEA_ affibodies. Immunohistochemistry was performed on formalin-fixed paraffin-embedded human gastric cancer tissue. The Z_CEA_ affibodies (Z_CEA_539, Z_CEA_546, and Z_CEA_919) stained tissue sections from human gastric cancer tissue [showed in brown color (upper layer)], which was similar to the staining pattern observed in polyclonal anti-CEA serum; nevertheless, human gastric cancer tissue stained with Z_WT_ and PBS did not exhibit any obvious signal (Figure 4). Furthermore, normal gastric tissue did not display any changes in sections incubated with Z_CEA_ affibodies and polyclonal anti-CEA serum (lower layer). Our result proved that Z_CEA_ affibodies bind specifically to native CEA expressed in the tumor tissue.

### 3.5 *In vivo* tumor-targeted imaging of Z_CEA_546 affibody

The *in vivo* biodistribution of Z_CEA_ affibodies is shown in Supplementary Figure S1. The Z_CEA_ affibodies were quickly distributed into most parts of the body within 30 min post-injection, excreted through the kidneys, and rapidly cleared from the body within 24 h.

**Figure 5:**
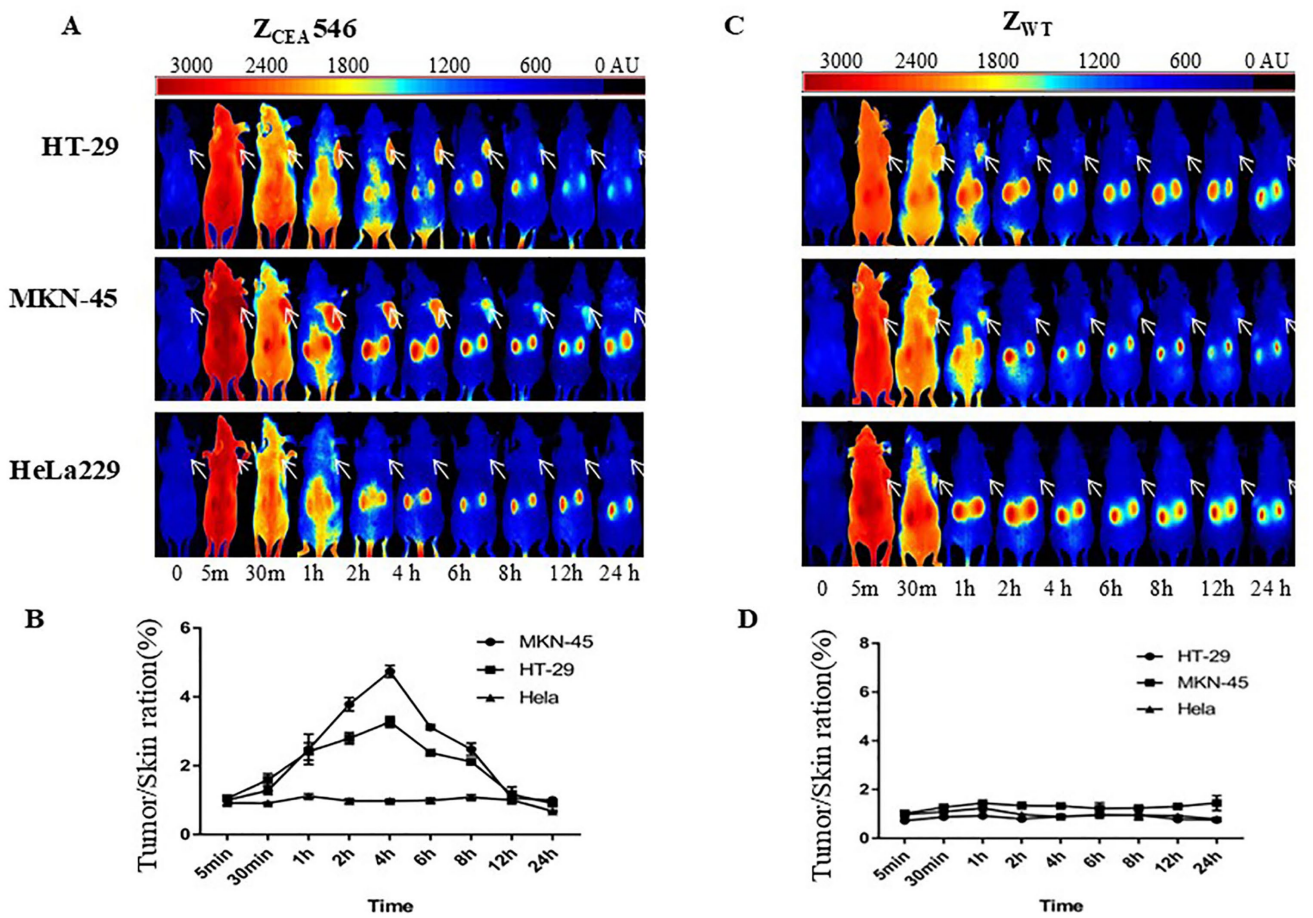
Tumor-targeting of Z_CEA_ affibodies. **(A)** *In vivo* fluorescence imaging of tumor-bearing mice (arrows) injected with Dylight755-conjugated Z_CEA_546 affibody at 0, 0.5, 30, 1, 2, 4, 6, 8, 12, and 24 h. **(B)** Tumor-to-background ratio of mice injected with Dylight755-conjugated Z_CEA_546 affibody. **(C)** *In vivo* fluorescence imaging of tumor-bearing mice (arrows) injected with Dylight755-conjugated Z_WT_ affibody at 0, 0.5, 30, 1, 2, 4, 6, 8, 12, and 24 h. **(D)** Tumor-to-background ratio of mice injected with Dylight755-conjugated Z_WT_ affibody. **(E)** The *in vivo* tumor targeting ability of Z_CEA_546 after 4 h post-injection compared to Z_WT_. Data are displayed as mean ± SD (*n* = 3). ^***^p < 0.001 compared to control.

We further explored the tumor targeting selectivity of Z_CEA_546 affibody in CEA tumor-bearing mice. After injection of DyLight755-labeled Z_CEA_546 into the tail vein of CEA-tumor bearing mice, fluorescence signals were observed at the site of the tumor 1 h after injection and maximum fluorescence intensity was observed at 4 h, which remained for 24 h (Figures 5A,B). However, in HeLa229 tumor-bearing mice, DyLight755-labeled Z_CEA_546 tumor-specific fluorescence signal could not be detected in the xenograft model, and DyLight755-labeled Z_WT_ also did not show fluorescence signal in xenograft tumor models (Figures 5C–E).

To verify whether *in vivo* fluorescence imaging retention is only present at the tumor site, the mice were sacrificed to isolate tumors and major organs for *ex-vivo* analysis at 24 h post-injection. After intravenous injection of DyLight755-labeled Z_CEA_546, strong fluorescence signals from the tumors derived from HT-29 and MK-45 cell lines were clearly detected; however, no obvious fluorescence signal was detected in HeLa229 cell line (Supplementary Figure S2).

## 4 Discussion and conclusion

Fluorescence imaging has displayed a high sensitivity and strong specificity with the advent of fluorescent probes combined with targeted ligands in recent years and could play a crucial role *in vivo* diagnosis of tumors and image-guided surgery. In particular, fluorescence imaging could be employed to reduce the high rate of missed diagnosis of gastrointestinal cancer and increase the outcomes of conventional surgery (Mieog et al., 2022; Stibbe et al., 2023). Numerous molecular imaging trials utilizing fluorescent probes targeting well-known tumor markers such as EGFR, VEGF, and HER2 have demonstrated potential to improve the diagnosis and treatment of gastrointestinal cancer (Lamberts et al., 2017; Terwisscha van Scheltinga et al., 2011; Goetz et al., 2013). However, these tumor markers are not universally overexpressed in gastrointestinal cancers. For instance, HER2 overexpression occurs in 3.8–36.6% of gastric cancers (Boku, 2014) and in 1.3–47.7% of colorectal cancers (CRCs) (Takegawa and Yonesaka, 2017). In addition, these targets are sometimes overexpressed in inflammatory tissues, resulting in reduced specificity. Therefore, the development of fluorescent molecular imaging techniques based on exclusive biomarkers of gastrointestinal tumors is urgently required.

CEA is overexpressed in almost all gastrointestinal cancers, especially in colorectal cancer, but its expression is limited in normal tissues (Emini et al., 1985). This highly specific expression of CEA in tumor tissues makes it one of the preferred biomarkers for targeting colorectal cancer. In a non-invasive imaging animal experiment, iodine-124 labeled antibodies to CEA have been used to enhance positron emission tomographic (PET) imaging of colorectal cancer (Schoffelen et al., 2012). Currently, phase III clinical trials have shown that the use of SGM-101, an anti-CEA antibody labeled with a fluorescent dye, improves detection and aids in the complete resection of CEA-positive tumors (Boogerd et al., 2018; de Valk et al., 2021). Fluorescent probes based on monoclonal antibodies have been extensively used for therapeutic, diagnostic, and biotechnological applications due to their high target specificity and binding affinity (Lamberts et al., 2017; Hernot et al., 2019). Nevertheless, monoclonal antibodies (mAb) continue to encounter several challenges in their clinical applications. Notably, their large size (~150 kDa) and insufficient tissue penetration results in poor vivo imaging diagnosis and treatment. Additionally, the presence of complex disulfide bonds and post-translational glycosylation modifications, along with the challenges of difficult and expensive production, have limited their clinical use (Ruigrok et al., 2011; Vazquez-Lombardi et al., 2015).

To overcome these drawbacks, non-immunoglobulin scaffolds have been reported as promising alternatives, such as affibodies, alphabodies, pronectins, affimers, centyrins, and repebodies (Škrlec et al., 2015). In this study, we describe the development and characterization of three novel affibodies (Z_CEA_539, Z_CEA_546, and Z_CEA_919) that selectively bind to CEA from a combinatorial affibody library. Subsequently, the Z_CEA_ affibodies were expressed in a prokaryotic expression system to explore the binding specificity to CEA. SPR assay demonstrated that the Z_CEA_ affibodies could bind to CEA with high affinity and specificity, yielding apparent KD values that were up to 10^6^ higher than the KD value of Z_WT_ (control) and reaching micromolar levels, consistent with previous reports on affibodies targeting EBV-latent membrane proteins (LMP) in our laboratory (Zhu S. et al., 2020; Kamara et al., 2021). Indirect immunofluorescence assay further proved the binding affinity and specificity of Z_CEA_ affibodies to tumor cells expressing CEA. Furthermore, immunohistochemistry staining showed that the three selected Z_CEA_ affibodies bind to the protein of interest (CEA) in human gastric cancer tissues. These results suggest that the Z_CEA_ affibodies selectively bind to CEA target protein with high binding affinity and specificity. Therefore, Z_CEA_ affibodies could be used for further fluorescence imaging of mice-bearing CEA cancer cell lines.

For molecular imaging, an ideal imaging probe should have a smaller molecular weight, exhibit high binding affinity and selectivity, ensure rapid biodistribution and tissue permeability, and be quickly cleared to achieve high-contrast imaging (Herschman, 2003). As a new class of binding molecule, affibodies can provide effective tumor penetration and high affinity. In preclinical and clinical studies, the HER2 binding affibody molecules have been developed and successfully tested as imaging tracers to detect HER2 overexpressed tumors, such as Z_HER2_:342 (Lee et al., 2008; Baum et al., 2010). A previous study comparing the performance of affibody Z_HER2:_342 with HER2-targeting scFv in tumor imaging showed that affibody molecules targeting HER2 provided increased uptake in tumor and a higher tumor-to-blood ratio (Orlova et al., 2006). In this study, right after 1 h of intravenous injection of DyLight755-labeled Z_CEA_546, a strong fluorescence signal was detected at the tumor site of the tumor-bearing mice, peaking at 4 h and maintaining fluorescence at that level for 24 h. Furthermore, dynamic biodistribution showed that DyLight755-labeled Z_CEA_546 was quickly distributed in the mouse body after injection, excreted from the kidney, and completely cleared from the body at 72 h. Therefore, DyLight755-labeled Z_CEA_546 affibody has great potential for use in molecular imaging diagnosis.

In conclusion, this study generated three novel affibodies (Z_CEA_539, Z_CEA_546, and Z_CEA_919), whose ability to bind CEA target protein was characterized through SPR, indirect immunofluorescence assay, and immunohistochemistry staining. Furthermore, DyLight755-labeled Z_CEA_546 displayed significant tumor targeting capability in CEA+ tumor cell line xenograft mice models (HT-29 and MKN-45). Thus, our results suggest that Z_CEA_ affibodies are promising tumor-specific fluorescent molecular imaging agents for detecting CEA expression in gastrointestinal cancers.

## Data Availability

The original contributions presented in the study are included in the article/Supplementary material, further inquiries can be directed to the corresponding authors.

## References

[ref1] Aavula S. M. Nimmagadda S. V. Biradhar N. Sula S. Chandran D. Lingala R.. (2011). Generation and characterization of an scFv directed against site II of rabies glycoprotein. Biotechnol. Res. Int. 2011, 652147, 1–652111. doi: 10.4061/2011/652147 22007309 PMC3189463

[ref2] Allemani C. Matsuda T. di Carlo V. Harewood R. Matz M. Nikšić M.. (2018). Global surveillance of trends in cancer survival 2000-14 (CONCORD-3): analysis of individual records for 37 513 025 patients diagnosed with one of 18 cancers from 322 population-based registries in 71 countries. Lancet 391, 1023–1075. doi: 10.1016/S0140-6736(17)33326-3, 29395269 PMC5879496

[ref3] Axon A. (2008). Is diagnostic and therapeutic endoscopy currently appropriate?: suggestions for improvement. Best Pract. Res. Clin. Gastroenterol. 22, 959–970. doi: 10.1016/j.bpg.2008.07.003, 18790441

[ref4] Baum R. P. Prasad V. Müller D. Schuchardt C. Orlova A. Wennborg A.. (2010). Molecular imaging of HER2-expressing malignant tumors in breast cancer patients using synthetic 111In-or 68Ga-labeled affibody molecules. J Nucl Med 51, 892–897. doi: 10.2967/jnumed.109.073239, 20484419

[ref5] Beauchemin N. Arabzadeh A. (2013). Carcinoembryonic antigen-related cell adhesion molecules (CEACAMs) in cancer progression and metastasis. Cancer Metastasis Rev. 32, 643–671. doi: 10.1007/s10555-013-9444-6, 23903773

[ref6] Beauchemin N. Benchimol S. Cournoyer D. Fuks A. Stanners C. P. (1987). Isolation and characterization of full-length functional cDNA clones for human carcinoembryonic antigen. Mol. Cell. Biol. 7, 3221–3230. doi: 10.1128/mcb.7.9.3221-3230.1987 3670312 PMC367958

[ref7] Boku N. (2014). HER2-positive gastric cancer. Gastric Cancer 17, 1–12. doi: 10.1007/s10120-013-0252-z, 23563986 PMC3889288

[ref8] Boogerd L. S. F. Hoogstins C. E. S. Schaap D. P. Kusters M. Handgraaf H. J. M. van der Valk M. J. M.. (2018). Safety and effectiveness of SGM-101, a fluorescent antibody targeting carcinoembryonic antigen, for intraoperative detection of colorectal cancer: a dose-escalation pilot study. Lancet Gastroenterol. Hepatol. 3, 181–191. doi: 10.1016/S2468-1253(17)30395-3, 29361435

[ref9] de Valk K. S. Deken M. M. Schaap D. P. Meijer R. P. Boogerd L. S. Hoogstins C. E.. (2021). Dose-finding study of a CEA-targeting agent, SGM-101, for intraoperative fluorescence imaging of colorectal cancer. Ann. Surg. Oncol. 28, 1832–1844. doi: 10.1245/s10434-020-09069-2, 33034788 PMC7892528

[ref10] Eidelman F. J. Fuks A. DeMarte L. Taheri M. Stanners C. P. (1993). Human carcinoembryonic antigen, an intercellular adhesion molecule, blocks fusion and differentiation of rat myoblasts. J. Cell Biol. 123, 467–475. doi: 10.1083/jcb.123.2.467, 8408226 PMC2119830

[ref11] Emini E. A. Hughes J. V. Perlow D. S. Boger J. (1985). Induction of hepatitis a virus-neutralizing antibody by a virus-specific synthetic peptide. J. Virol. 55, 836–839. doi: 10.1128/jvi.55.3.836-839.1985, 2991600 PMC255070

[ref12] Fedorova A. Zobel K. Gill H. S. Ogasawara A. Flores J. E. Tinianow J. N.. (2011). The development of peptide-based tools for the analysis of angiogenesis. Chem. Biol. 18, 839–845. doi: 10.1016/j.chembiol.2011.05.011, 21802005

[ref13] Garnier J. Gibrat J. F. Robson B. (1996). GOR method for predicting protein secondary structure from amino acid sequence. Methods Enzymol. 266, 540–553. doi: 10.1016/S0076-6879(96)66034-0 8743705

[ref14] Garousi J. Andersson K. G. Mitran B. Pichl M. L. Ståhl S. Orlova A.. (2016). PET imaging of epidermal growth factor receptor expression in tumours using 89Zr-labelled ZEGFR:2377 affibody molecules. Int. J. Oncol. 48, 1325–1332. doi: 10.3892/ijo.2016.3369, 26847636 PMC4777594

[ref15] Geourjon C. Deléage G. (1995). SOPMA: significant improvements in protein secondary structure prediction by consensus prediction from multiple alignments. Comput. Appl. Biosci. 11, 681–684. doi: 10.1093/bioinformatics/11.6.681 8808585

[ref16] Goetz M. Hoetker M. S. Diken M. Galle P. R. Kiesslich R. (2013). In vivo molecular imaging with cetuximab, an anti-EGFR antibody, for prediction of response in xenograft models of human colorectal cancer. Endoscopy 45, 469–477. doi: 10.1055/s-0032-1326361, 23580409

[ref17] Gold P. Freedman S. O. (1965). Specific carcinoembryonic antigens of the human digestive system. J. Exp. Med. 122, 467–481. doi: 10.1084/jem.122.3.467, 4953873 PMC2138078

[ref18] Hefta S. A. Hefta L. J. Lee T. D. Paxton R. J. Shively J. E. (1988). Carcinoembryonic antigen is anchored to membranes by covalent attachment to a glycosylphosphatidylinositol moiety: identification of the ethanolamine linkage site. Proc. Natl. Acad. Sci. U.S.A. 85, 4648–4652. doi: 10.1073/pnas.85.13.4648, 3387431 PMC280492

[ref19] Hernot S. van Manen L. Debie P. Mieog J. S. D. Vahrmeijer A. L. (2019). Latest developments in molecular tracers for fluorescence image-guided cancer surgery. Lancet Oncol. 20, e354–e367. doi: 10.1016/S1470-2045(19)30317-1 31267970

[ref20] Herschman H. R. (2003). Molecular imaging: Looking at problems, seeing solutions. Science 302, 605–608. doi: 10.1126/science.1090585 14576425

[ref21] Hopp T. P. Woods K. R. (1981). Prediction of protein antigenic determinants from amino acid sequences. Proc. Natl. Acad. Sci. U.S.A. 78, 3824–3828. doi: 10.1073/pnas.78.6.3824 6167991 PMC319665

[ref22] Ilantzis C. DeMarte L. Screaton R. A. Stanners C. P. (2002). Deregulated expression of the human tumor marker CEA and CEA family member CEACAM6 disrupts tissue architecture and blocks colonocyte differentiation. Neoplasia 4, 151–163. doi: 10.1038/sj.neo.7900201 11896570 PMC1550325

[ref23] Jameson B. A. Wolf H. (1988). The antigenic index: a novel algorithm for predicting antigenic determinants. Comput. Appl. Biosci. 4, 181–186. doi: 10.1093/bioinformatics/4.1.181, 2454713

[ref24] Kamara S. Guo Y. Mao S. Ye X. Li Q. Zheng M.. (2021). Novel EBV LMP1 C-terminal domain binding affibody molecules as potential agents for in vivo molecular imaging diagnosis of nasopharyngeal carcinoma. Appl. Microbiol. Biotechnol. 105, 7283–7293. doi: 10.1007/s00253-021-11559-6, 34505914

[ref25] Lamberts L. E. Koch M. de Jong J. S. Adams A. L. L. Glatz J. Kranendonk M. E. G.. (2017). Tumor-specific uptake of fluorescent bevacizumab-IRDye800CW microdosing in patients with primary breast Cancer: a phase I feasibility study. Clin. Cancer Res. 23, 2730–2741. doi: 10.1158/1078-0432.CCR-16-0437 28119364

[ref26] Lee S. B. Hassan M. Fisher R. Chertov O. Chernomordik V. Kramer-Marek G.. (2008). Affibody molecules for *in vivo* characterization of HER2-positive tumors by near-infrared imaging. Clin. Cancer Res. 14, 3840–3849. doi: 10.1158/1078-0432.CCR-07-4076 18559604 PMC3398736

[ref27] Mieog J. S. D. Achterberg F. B. Zlitni A. Hutteman M. Burggraaf J. Swijnenburg R. J.. (2022). Fundamentals and developments in fluorescence-guided cancer surgery. Nat. Rev. Clin. Oncol. 19, 9–22. doi: 10.1038/s41571-021-00548-3 34493858

[ref28] Nolan K. F. Yun C. O. Akamatsu Y. Murphy J. C. Leung S. O. Beecham E. J.. (1999). Bypassing immunization: optimized design of “designer T cells” against carcinoembryonic antigen (CEA)-expressing tumors, and lack of suppression by soluble CEA. Clin. Cancer Res. 5, 3928–3941. 10632322

[ref29] Nord K. Nilsson J. Nilsson B. Uhlén M. Nygren P. A. (1995). A combinatorial library of an alpha-helical bacterial receptor domain. Protein Eng. 8, 601–608. doi: 10.1093/protein/8.6.601, 8532685

[ref30] Ordoñez C. Screaton R. A. Ilantzis C. Stanners C. P. (2000). Human carcinoembryonic antigen functions as a general inhibitor of anoikis. Cancer Res. 60, 3419–3424, 10910050

[ref31] Orlova A. Magnusson M. Eriksson T. L. Nilsson M. Larsson B. Höidén-Guthenberg I.. (2006). Tumor imaging using a picomolar affinity HER2 binding affibody molecule. Cancer Res. 66, 4339–4348. doi: 10.1158/0008-5472.CAN-05-3521, 16618759

[ref32] Ruigrok V. J. Levisson M. Eppink M. H. Smidt H. van der Oost J. (2011). Alternative affinity tools: more attractive than antibodies? Biochem. J. 436, 1–13. doi: 10.1042/BJ20101860 21524274

[ref33] Schardt J. S. Oubaid J. M. Williams S. C. Howard J. L. Aloimonos C. M. Bookstaver M. L.. (2017). Engineered multivalency enhances affibody-based HER3 inhibition and downregulation in cancer cells. Mol. Pharm. 14, 1047–1056. doi: 10.1021/acs.molpharmaceut.6b00919, 28248115 PMC5433087

[ref34] Schlick C. J. R. Khorfan R. Odell D. D. Merkow R. P. Bentrem D. J. (2020). Margin positivity in resectable esophageal cancer: are there modifiable risk factors? Ann. Surg. Oncol. 27, 1496–1507. doi: 10.1245/s10434-019-08176-z, 31933223 PMC7321808

[ref35] Schoffelen R. van der Graaf W. T. Sharkey R. M. Franssen G. M. McBride W. J. Chang C. H.. (2012). Pretargeted immuno-PET of CEA-expressing intraperitoneal human colonic tumor xenografts: a new sensitive detection method. EJNMMI Res. 2:5. doi: 10.1186/2191-219X-2-5, 22284761 PMC3298693

[ref36] Shah K. Weissleder R. (2005). Molecular optical imaging: applications leading to the development of present day therapeutics. NeuroRx 2, 215–225. doi: 10.1602/neurorx.2.2.215, 15897946 PMC1064987

[ref37] Škrlec K. Štrukelj B. Berlec A. (2015). Non-immunoglobulin scaffolds: a focus on their targets. Trends Biotechnol. 33, 408–418. doi: 10.1016/j.tibtech.2015.03.012, 25931178

[ref38] Ståhl S. Gräslund T. Eriksson Karlström A. Frejd F. Y. Nygren P. Å. Löfblom J. (2017). Affibody molecules in biotechnological and medical applications. Trends Biotechnol. 35, 691–712. doi: 10.1016/j.tibtech.2017.04.007, 28514998

[ref39] Stibbe J. A. Hoogland P. Achterberg F. B. Holman D. R. Sojwal R. S. Burggraaf J.. (2023). Highlighting the undetectable - fluorescence molecular imaging in gastrointestinal endoscopy. Mol. Imaging Biol. 25, 18–35. doi: 10.1007/s11307-022-01741-1 35764908 PMC9971088

[ref40] Sung H. Ferlay J. Siegel R. L. Laversanne M. Soerjomataram I. Jemal A.. (2021). Global cancer statistics 2020: GLOBOCAN estimates of incidence and mortality worldwide for 36 cancers in 185 countries. CA Cancer J. Clin. 71, 209–249. doi: 10.3322/caac.21660, 33538338

[ref41] Takegawa N. Yonesaka K. (2017). HER2 as an emerging oncotarget for colorectal Cancer treatment after failure of anti-epidermal growth factor receptor therapy. Clin. Colorectal Cancer 16, 247–251. doi: 10.1016/j.clcc.2017.03.001, 28363756

[ref42] Terwisscha van Scheltinga A. G. van Dam G. M. Nagengast W. B. Ntziachristos V. Hollema H. Herek J. L.. (2011). Intraoperative near-infrared fluorescence tumor imaging with vascular endothelial growth factor and human epidermal growth factor receptor 2 targeting antibodies. J Nucl Med 52, 1778–1785. doi: 10.2967/jnumed.111.092833 21990576

[ref43] Thompson J. A. Pande H. Paxton R. J. Shively L. Padma A. Simmer R. L.. (1987). Molecular cloning of a gene belonging to the carcinoembryonic antigen gene family and discussion of a domain model. Proc. Natl. Acad. Sci. U.S.A. 84, 2965–2969. doi: 10.1073/pnas.84.9.2965, 3033672 PMC304781

[ref44] Uhlén M. Guss B. Nilsson B. Gatenbeck S. Philipson L. Lindberg M. (1984). Complete sequence of the staphylococcal gene encoding protein a. a gene evolved through multiple duplications. J. Biol. Chem. 259, 1695–1702. doi: 10.1016/S0021-9258(17)43463-6, 6319407

[ref45] Vazquez-Lombardi R. Phan T. G. Zimmermann C. Lowe D. Jermutus L. Christ D. (2015). Challenges and opportunities for non-antibody scaffold drugs. Drug Discov. Today 20, 1271–1283. doi: 10.1016/j.drudis.2015.09.004 26360055

[ref46] Wang D. Li R. Jiang J. Qian H. Xu W. (2023). Exosomal circRNAs: novel biomarkers and therapeutic targets for gastrointestinal tumors. Biomed. Pharmacother. 157:114053. doi: 10.1016/j.biopha.2022.114053, 36462315

[ref47] Xue X. Wang B. Du W. Zhang C. Song Y. Cai Y.. (2016). Generation of affibody molecules specific for HPV16 E7 recognition. Oncotarget 7, 73995–74005. doi: 10.18632/oncotarget.12174, 27659535 PMC5342030

[ref48] Zhang Q. Wang F. Chen Z. Y. Wang Z. Zhi F. C. Liu S. D.. (2016). Comparison of the diagnostic efficacy of white light endoscopy and magnifying endoscopy with narrow band imaging for early gastric cancer: a meta-analysis. Gastric Cancer 19, 543–552. doi: 10.1007/s10120-015-0500-5, 25920526

[ref49] Zhu S. Chen J. Xiong Y. Kamara S. Gu M. Tang W.. (2020). Novel EBV LMP-2-affibody and affitoxin in molecular imaging and targeted therapy of nasopharyngeal carcinoma. PLoS Pathog. 16:e1008223. doi: 10.1371/journal.ppat.1008223, 31905218 PMC6964910

[ref50] Zhu J. Kamara S. Cen D. Tang W. Gu M. Ci X.. (2020). Generation of novel affibody molecules targeting the EBV LMP2A N-terminal domain with inhibiting effects on the proliferation of nasopharyngeal carcinoma cells. Cell Death Dis. 11:213. doi: 10.1038/s41419-020-2410-7, 32238802 PMC7113277

[ref51] Zhu J. Kamara S. Wang Q. Guo Y. Li Q. Wang L.. (2021). Novel affibody molecules targeting the HPV16 E6 oncoprotein inhibited the proliferation of cervical cancer cells. Front Cell Dev Biol 9:677867:677867. doi: 10.3389/fcell.2021.677867, 34109181 PMC8181454

[ref52] Zimmerman J. M. Eliezer N. Simha R. (1968). The characterization of amino acid sequences in proteins by statistical methods. J. Theor. Biol. 21, 170–201. doi: 10.1016/0022-5193(68)90069-6 5700434

[ref53] Zimmermann W. Ortlieb B. Friedrich R. von Kleist S. (1987). Isolation and characterization of cDNA clones encoding the human carcinoembryonic antigen reveal a highly conserved repeating structure. Proc. Natl. Acad. Sci. U.S.A. 84, 2960–2964. doi: 10.1073/pnas.84.9.2960 3033671 PMC304780

