# Correction: Novel anti-CEA affibody for rapid tumor-targeting and molecular imaging diagnosis in mice bearing gastrointestinal cancer cell lines

**DOI:** 10.3389/fmicb.2026.1788283

**Published:** 2026-04-10

**Authors:** Huanyi Shao, Kaiji Lv, Pengfei Wang, Jinji Jin, Yiqi Cai, Jun Chen, Saidu Kamara, Shanli Zhu, Guanbao Zhu, Lifang Zhang

**Affiliations:** 1 Department of Pediatric Surgery, The First Affiliated Hospital of Wenzhou Medical University, Wenzhou, China; 2 Department of Microbiology and Immunology, School of Basic Medical Sciences, Institute of Molecular Virology and Immunology, Wenzhou Medical University, Wenzhou, China; 3 Department of Gastrointestinal Surgery, The First Affiliated Hospital of Wenzhou Medical University, Wenzhou, China

**Keywords:** affibody molecules, gastrointestinal cancer, CEA, molecular imaging, tumor diagnosis

There was a mistake in Figure 3 as published. We have identified that during figure assembly an incorrect image was inadvertently placed in Figure 3B. The ZCEA919 affibody image was reused on the first panel of the ZCEA539 affibody in the published Figure 3B in the MKN-45 cell line. The duplication does not affect the conclusion of the study.

The corrected Figure 3 appears below.

**Figure 3:**
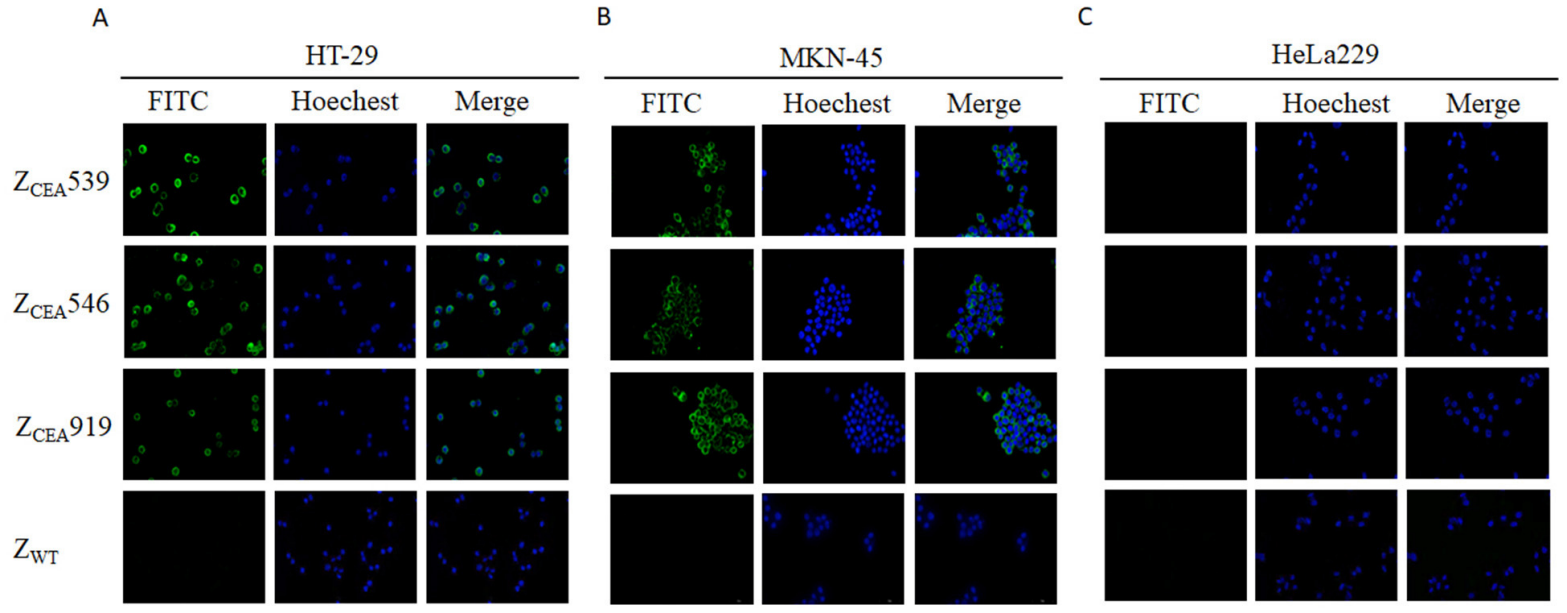
Binding of Z_CEA_ affibodies to HT-29 and MKN-45 cells by indirect immunofluorescence assay. **(A)** HT-29 **(B)** MKN-45 cells were incubated with Z_CEA_ affibodies. Mouse-anti-His-tag mAb FITC (green) was used to visualize the binding of Z_CEA_ affibodies to CEA+ cancer cell lines and nuclei were counterstained with Hoechst 3342 (400 × ). **(C)** HeLa229 served as the CEA cancer cell line. Z_WT_ set as the affibody negative control. Scale bar = 10 μm. Experiments were performed in triplicate.

The original version of this article has been updated.

