# Supplementary material for: Novel anti-CEA affibody for rapid tumor-targeting and molecular imaging diagnosis in mice bearing gastrointestinal cancer cell lines
Source: Front Microbiol. 2024 Oct 9;15:1464088. doi: 10.3389/fmicb.2024.1464088 (PMC11496145; doi:10.3389/fmicb.2024.1464088)
Supplement: Supplementary file 1 [file Data_Sheet_1.docx]

**Supplementary Material**

**Journal:**

**Frontiers in Microbiology**

**Title:**

Novel Anti-CEA Affibody for Rapid Tumor-Targeting and Molecular Imaging Diagnosis in Mice bearing Gastrointestinal cancer cell lines

**Authors:**

Huanyi Shao^1,3*^, Kaiji Lv^2*^, Pengfei Wang^2^, Jingji Jin^2^, [Yiqi Cai](https://pubmed.ncbi.nlm.nih.gov/?term=Cai+Y&cauthor_id=37586454)^2^, Jun Chen^3^, Saidu Kamara^3^, Shanli Zhu^3^, Guanbao Zhu^2#^, Lifang Zhang^3#^,

*These authors contributed equally to this work.

1. Department of Pediatric Surgery, The First Affiliated Hospital of

Wenzhou Medical University, Wenzhou 325000, Zhejiang, PR China

2. Department of Gastrointestinal Surgery, The First Affiliated Hospital

of Wenzhou Medical University, Wenzhou, Zhejiang, 325000, PR China

3. Institute of Molecular Virology and Immunology, Department of

Microbiology and Immunology, School of Basic Medical Sciences,

Wenzhou Medical University, Wenzhou 32503, Zhejiang, PR China

**#Corresponding authors**: Lifang Zhang ([wenzhouzlf@126.com](mailto:wenzhouzlf@126.com)), +86-577-86689910, Guanbao Zhu (zgbwmc@126.com)

**Table S1. Kinetic data from the SPR binding affinity analysis of the affibody**

**molecules**

| Affibody | KD |
| --- | --- |
| Z_CEA_539 | 5.059 x 10-6 mol/L |
| Z_CEA_546 | 1.733 x 10-7 mol/L |
| Z_CEA_919 | 5.512 x 10-6 mol/L |
| ZWT | 1.628 mol/L, |

Abbreviation: KD, Dissociation equilibrium constant


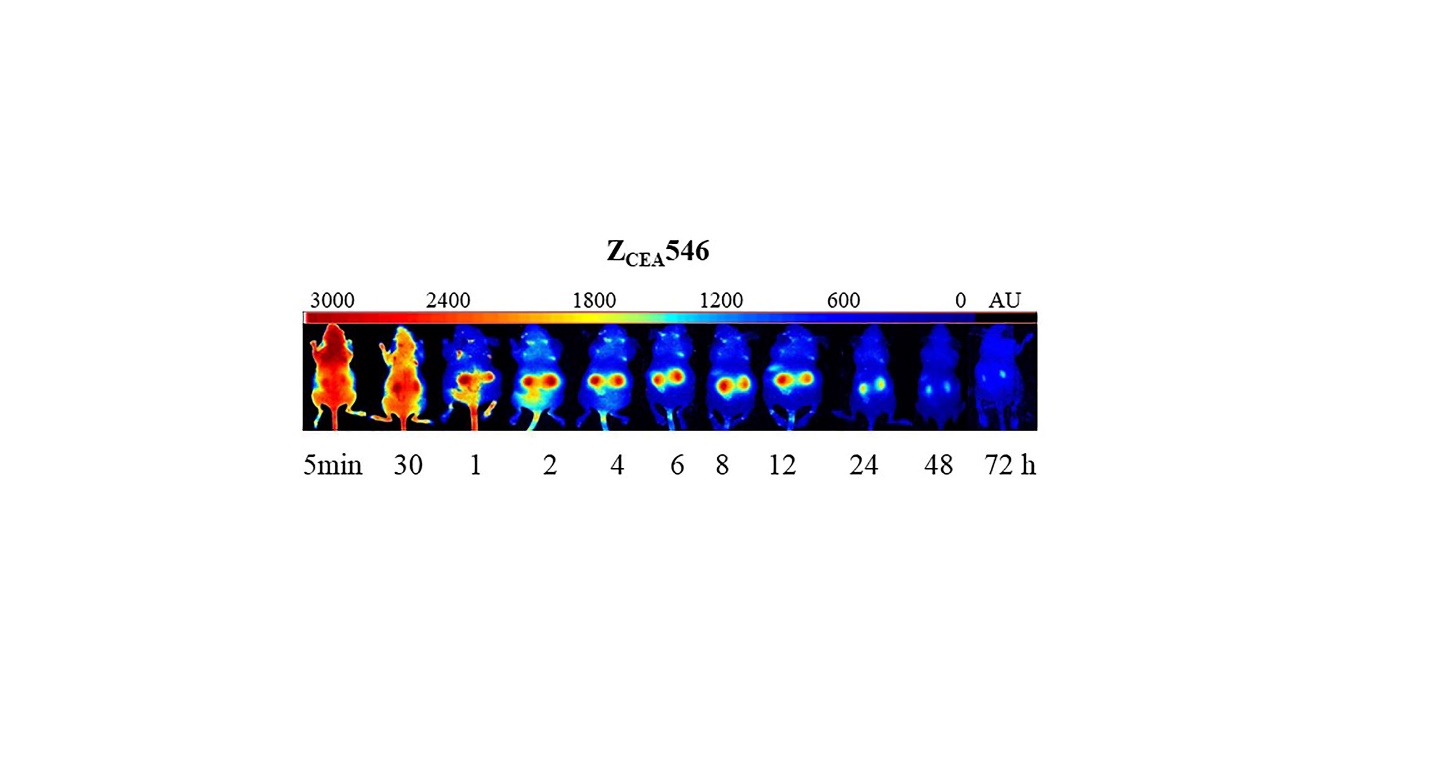


**Figure S1: In vivo biodistribution of Z_CEA_546 affibody molecules in healthy nude mice**. The labelled affibody molecules were confirmed by SDS-PAGE and further detected at wavelengths of 730-950 nm by an in vivo fluorescence imaging system. After tail vein injection with Dylight 755-labelled affibody molecules, fluorescence images were obtained from mice at different time points. Kidney uptake was prominent for the accumulation of affibody molecules. The accumulation of affibody molecules maximally occurred at 8 h after injection and then decreased over the time course. The signal was undetectable at 72 h.

**
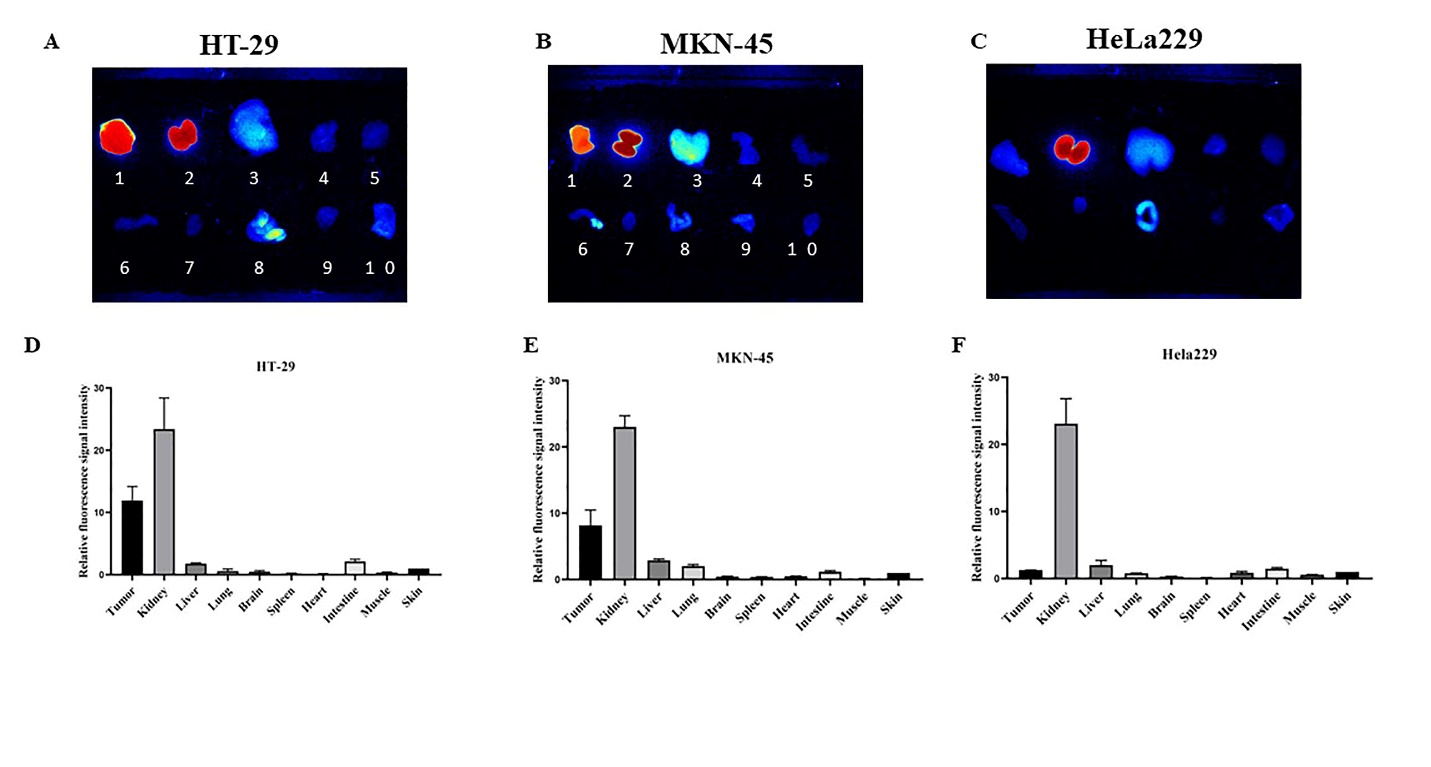
**

**Figure S2:** **Ex vivo imaging of the fluorescence retention of** **DyLight755-labeled** **Z_CEA_546 affibody in different tissues**. (A, B, C) Fluorescence imaging of tumor ex vivo at 24 h p.i. of DyLight755-labeled Z_CEA_546 affibody injection. In each image: 1, tumor; 2, kidney; 3, liver; 4, lung; 5, brain; 6, spleen; 7, heart; 8, intestine; 9, muscle; 10, skin. (D, E, F) The relative fluorescence signal intensity of ex vivo imaging in the tumors and major organs at 24 h p.i. of DyLight755-labeled injection. The value fluorescence signal intensity in the tumors and other major organs/fluorescence signal intensity in the corresponding muscle tissue
